# The "GfI-Fischartenatlas" of the German Ichthyological Society (GfI) - A compilation of validated fish species occurrence records

**DOI:** 10.3897/BDJ.14.e201405

**Published:** 2026-07-16

**Authors:** Heiko Brunken, Lars Braubach, Thore Engel, Peter Grobe, Claas-Thido Pfaff, Birgit Rach, Heide-Rose Vatterrott, Martin Friedrichs-Manthey

**Affiliations:** 1 Hochschule Bremen, Bremen, Germany Hochschule Bremen Bremen Germany; 2 Friedrich-Schiller Universität Jena, Jena, Germany Friedrich-Schiller Universität Jena Jena Germany; 3 German German Centre for Integrative Biodiversity Research (iDiv) Halle-Jena-Leipzig, Leipzig, Germany German German Centre for Integrative Biodiversity Research (iDiv) Halle-Jena-Leipzig Leipzig Germany; 4 Leibniz Institute for the Analysis of Biodiversity Change (LIB), Bonn, Germany Leibniz Institute for the Analysis of Biodiversity Change (LIB) Bonn Germany; 5 gfbio.org, Bremen, Germany gfbio.org Bremen Germany; 6 Helmholtz-Centre for Environmental Research - UFZ, Leipzig, Germany Helmholtz-Centre for Environmental Research - UFZ Leipzig Germany

## Abstract

**Background:**

Despite the urgent need for conservation, reliable data on the distribution of fish species across countries and federal states is limited for both freshwater and marine habitats. The German Ichthyological Society (Gesellschaft für Ichthyologie e.V. or GfI) aims to compile fish distribution data with a focus on Germany (including its marine areas), Austria and the marine areas of the Netherlands and Denmark. These data are openly available on the "GfI-Fischartenatlas" web portal which serves as an information platform at the intersection of science, practice and environmental education, promoting the conservation of fish biodiversity and their habitats. The GfI-Fischartenatlas is a joint project of the GfI and the City University of Applied Sciences Bremen. It was developed in cooperation between the biology and computer science departments at the university. The IT basis for collecting, managing and presenting the data online is the “Biodiversity Warehouse” software architecture. The data are hosted by the Biodiversity Informatics Department at the Museum Koenig Bonn, which is part of the Leibniz Institute for the Analysis of Biodiversity Change (LIB).

**New information:**

The GfI-Fischartenatlas consolidates fish distribution data from various cross-border sources into a unified platform. This includes both data collected specifically for the atlas and data integrated from other databases (in total n = 136,714, as of 13.03.2026). The data primary to the atlas are published as a dataset on the Global Biodiversity Information Facility (GBIF). This dataset (ABCD archive) contains occurrence records (n = 62,545) for 264 freshwater and marine fish species, derived from scientific and grey literature as well as citizen0science observations, covering 16 European countries (with a focus on Germany and Austria).

## Introduction

To date, over 36,000 fish species have been described globally (Froese and Pauly 2025), yet, data on their occurrence and conservation status are scarce (Maasri et al. 2022). Both freshwater and marine fish species are under immense pressure from various threats, such as overfishing, pollution, climate change and invasive species (Sumaila and Tai 2020, Vardakas et al. 2025). As a consequence, a large proportion of all fish species is considered threatened by the IUCN (IUCN 2023). In Europe, at least 37% of freshwater fish are considered extinct or endangered (Brooks and Freyhof 2011) with an additional 5.3% classified as data deficient. For marine fish species, 10% are considered "endangered" or "potentially endangered", plus another 20.6% of marine fish species are classified as "data deficient" (Nieto et al. 2015).

Fish species conservation is largely hindered by the availability of relevant data for the majority of species (Maasri et al. 2022). With the exception of some species that were either charismatic or relevant economically or as a food source (e.g. diverse sturgeon species, Haidvogl et al. (2015)), we largely miss historical data sources for long-term assessments. In Europe, the amount of data has increased recently, partly due to legal regulations that require regular monitoring, such as the Water Framework Directive (European Parliament and Council 2000) and the Habitats Directive (Council of the European Communities 1992) for freshwater habitats and the Marine Strategy Framework Directive for marine fish stocks (European Parliament and Council 2008). However, due to competencies organised on a country or even federal level, such data are often scattered and, furthermore, data availability is often hindered by technical and legal obstacles. In addition, the pronounced "data wave" from citizen-science projects, which has dramatically improved data availability for a wide range of diverse taxa, has largely passed fish species, likely because of difficulties in observing and identifying species underwater.

To close data gaps and increase availability, particularly of historical occurrence data, the City University of Applied Sciences Bremen and the German Ichthyological Society (Gesellschaft für Ichthyologie e.V., GfI) launched the Fischartenatlas für Deutschland (Fish Species Atlas for Germany) in 2006 (Brunken and Brunschön 2006). The database contains fish occurrence data compiled from heterogeneous data sources, including scientific papers, regional atlases, grey literature and the personal data collections of society members and other registered users. Since its launch, the database has undergone continuous development in collaboration with the biology and computer science departments at the City University of Applied Sciences Bremen (Brunken et al. 2012, Steinhausen et al. 2012,Brunken and Vatterrott 2019, Mehrhoff et al. 2021). In 2007, due to a collaboration with the University of Salzburg, the Fischartenatlas was further developed into the binational Fish Species Atlas of Germany and Austria (later Fischfauna-Online, from here on called "GfI-Fischartenatlas"). Recently, marine fish were added for German coastal waters and the German Exclusive Economic Zone (EEZ), as well as for the area covered by the Trilateral Wadden Sea Cooperation in the Netherlands, Germany and Denmark.

However, due to its long-term development and varied data provenance, it was unclear whether and how the database could be shared openly, for example, on GBIF. Recently, a collaboration with the German National Research Data Infrastructure for Biodiversity (NFDI4Biodiversity) clarified the legal requirements for data sharing (Grünberger 2025). The report provides an introduction to several relevant sub-aspects: copyright, database rights, data protection, research ethics and licensing. In summary, the transfer of the compiled GfI dataset to GBIF is generally permissible, as individual species occurrence data do not enjoy copyright protection (with the exception of photographic evidence). However, while the principles of good scientific practice encourage the inclusion of names and institutional affiliations of voluntary contributors within the metadata, such disclosure remains subject to individual consent for data protection reasons. The GfI-Fischartenatlas data were published in accordance with the recommendations provided by Grünberger (2025).

## General description

### Purpose

**Figure 1.**
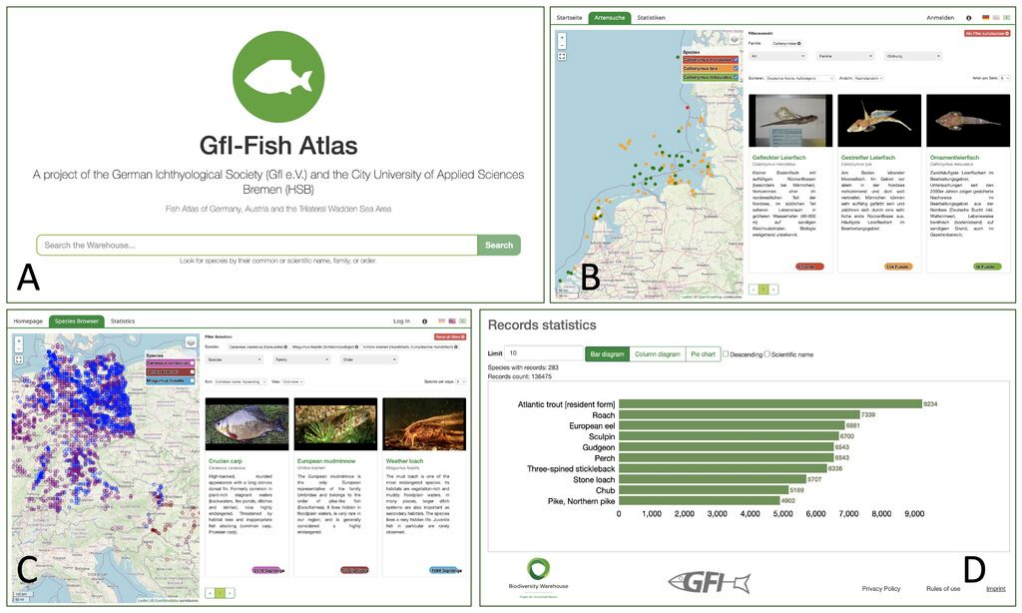
GfI-Fischartenatlas. **A** Home page with search bar; **B** Result of family search Callionymidae (display in German); **C** Result of species search *Carassius carassius*, *Umbra krameri* and *Misgurnus fossilis* (display in English); **D** Statistics - Number of records per species.

**Figure 2.**
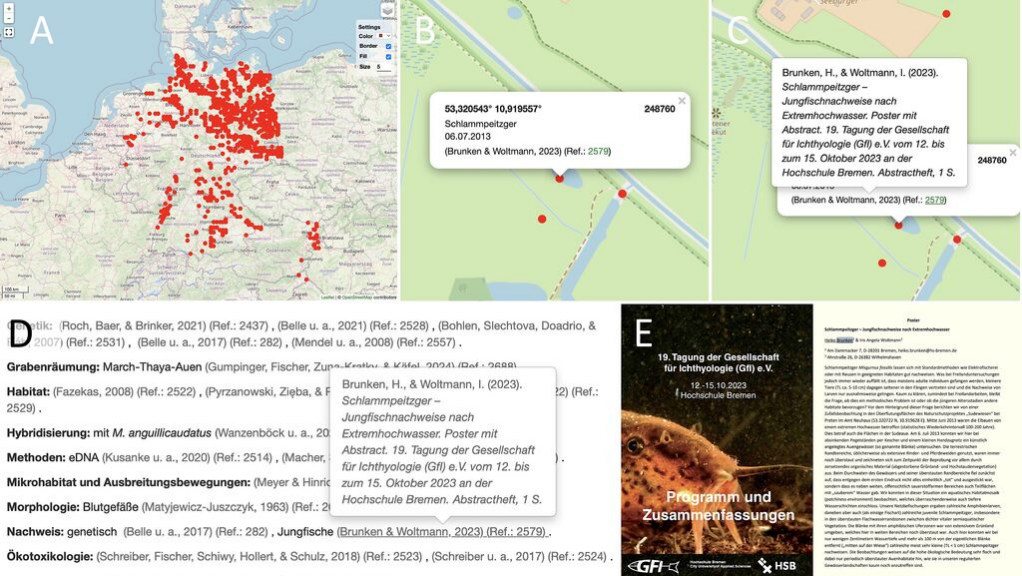
GfI-Fischartenatlas. From distribution point (record) to the original source: **A** Distribution map; **B** Short citation; **C** Full citation; **D** Bibliography with link to the original source; **E** Original source.

As a zoological society specialising in fish, one of the aims of the German Ichthyological Society (GfI) is to create a German-language forum for information, communication and scientific exchange in the field of fish science (Ichthyology). In addition, the GfI strives to contribute to the protection of fish species and their habitats. A key project is the GfI-Fischartenatlas (Brunken et al. 2026, Brunken and Vatterrott 2026) (Figs 1, 2), a data portal, aiming to report on ichthyological diversity and science. The GfI-Fischartenatlas also serves as a science-policy interface. The data are freely available and contains distribution maps and species descriptions of all freshwater fish species in Germany and Austria, as well as marine species from adjacent waters. The species descriptions include detailed information on the species' taxonomy, systematics, morphology and ecology and are supplemented by photos and literature references. The GfI-Fischartenatlas is designed to be trilingual (German, English and Portuguese).

**Figure 3.**
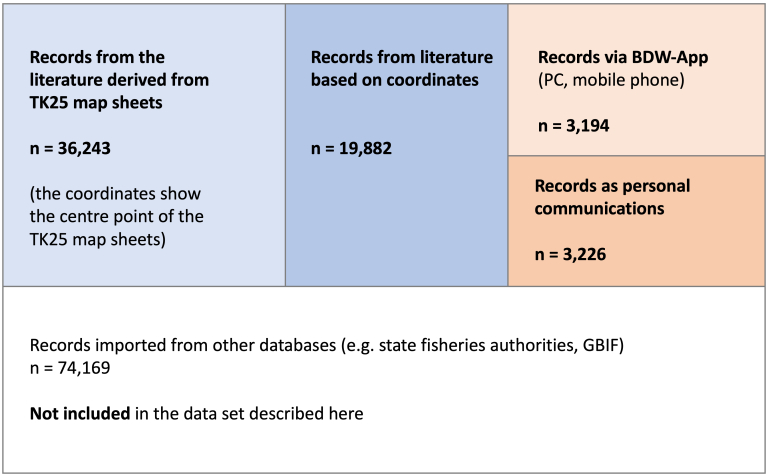
Data sources by reference type. The data exported to GBIF includes the fields highlighted in blue and orange (n = 62,545). Records via BDW-App = own notification.

Numbers of *records* (as of 13.03.2026) (Fig. 3):

ABCD archive records in total: n = 62,545

- Literature data are based on records derived from TK25 map sheets (German 1:25,000 topographic maps), such as those found in state-level fish species registers : n = 36,243;
- Literature data with exact coordinates: n = 19,882;
- Data referenced as "Personal communication": n = 3,226;
- Data referenced as "Own Notification": n = 3,194.

## Project description

### Personnel

The development of the GfI-Fischartenatlas is primarily led by faculty members at the City University of Applied Sciences Bremen and is fully integrated into the curriculum. The development is designed as a university teaching module that offers clear added value to students by being closely aligned with real-world practice examples. Embedded within the degree programmes in Biology and Computer Science, the module following the principles of research-based learning. In biology, this enables students to combine theoretical knowledge of biodiversity with hands-on methodological skills in data collection, as well as the subsequent use of collected data on digital platforms. Computer science students, in turn, gain practical experience in addressing the complex technical demands of data processing and in engaging in interdisciplinary communication. By linking research and learning outcomes to a real-world project with significant relevance to nature conservation and species protection, the module provides strong motivational incentives for students. This example illustrates how such integration of research, teaching and practical application offers substantial educational benefits. Moreover, this approach holds considerable potential for transfer and could be effectively adapted to other university contexts and disciplinary settings.

### Funding

The GfI-Fischartenatlas is hosted by the Leibniz Institute for the Analysis of Biodiversity Change (LIB) - Museum Koenig, Bonn. The project receives no further financial support from third parties. Both the biological and IT content were developed without any public funding, as part of research-based learning and through the voluntary work of the German Ichthyological Society (GfI e.V.). The software architecture is developed exclusively as part of the courses in the Master's degree programme "Complex Software Systems".

## Sampling methods

### Study extent

Data are continuously compiled as part of the routine scientific and ichthyological work conducted at the City University of Applied Sciences Bremen and within the German Ichthyological Society, notably through networking on scientific online platforms, such as ResearchGate and Academia.edu. These data are completed by targeted searches in regional natural history or conservation-orientated journals, which are often less accessible through major scientific search engines. Finally, grey literature, including environmental reports and various academic theses, is scanned and included in the GFI-Fischartentals. In rare occasions, occurrence data from press articles or other online media sources are included after careful validation. Consequently, all data in the GfI-Fischartenatlas are presence-only information.

Species taxonomy from the aformentioned diverse data sources is mapped to the Eschmeyer’s Catalogue of Fishes (Fricke et al. 2025), which serves as the taxonomic reference database.

### Sampling description

**Figure 4.**
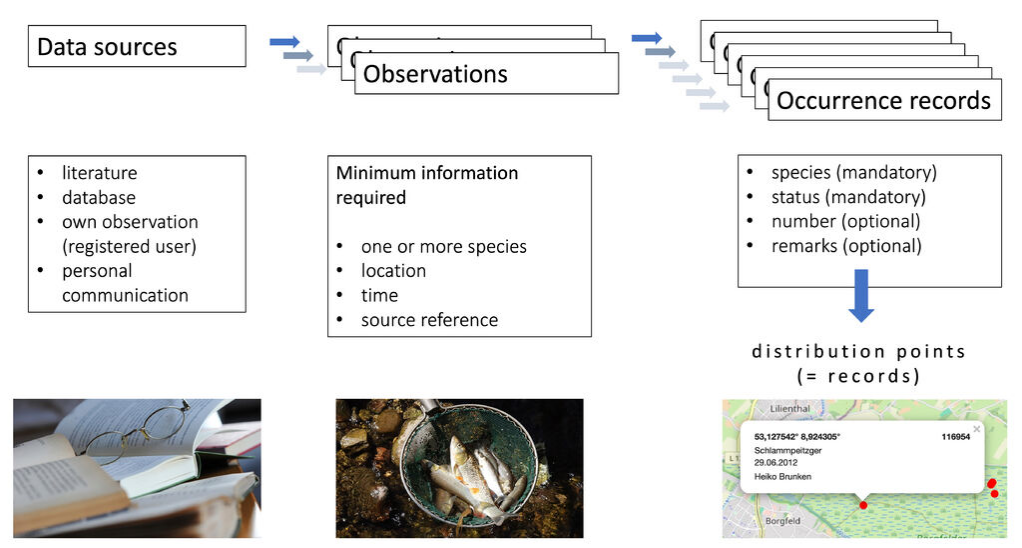
GfI-Fischartenatlas. From source to distribution point (record) in map.

A record in the GfI-Fischartenatlas is obtained as follows:

One *source* (e.g. publication) can contain several *observations*, for example, the results of several sampling events. Each *observation* (same time, same place, same source, ...) can, in turn, contain one or more species. For each species, a *distribution point* (= *record*) is generated in the GfI-Fischartenatlas (Fig. 4). Species-specific information (e.g. status, number) can be managed for each of the species, additionally.

Each record contains information on: (1)* Species Name (drop-down list); (2) Species Number (number); (3)* Species Status (drop-down list); (4) Species Comment (text); (5)* Location Coordinates; (6) Location Concealed (click box); (7) Location Format (DD or DMS); (8) Location Reference System (e.g. WGS84); (9) Location Deviation [m]; (10) Location Comment; (11)* Date from (date); (12) Date to (date); (13)* Survey Method (drop-down list); (14) Comment; (15)* Reference Type (drop-down list); (16) Image(s) (upload); (17) Rights (click boxes). * indicates mandatory entries.

(3): Status information is distinguished with regard to developmental stage (adult, subadult, juvenile) and condition (alive, dead record).

(13): With regard to the survey methods for recording fish fauna mentioned in the references, the BDW database distinguishes between the following categories (mandatory information): (0) No information; (1) Angling; (2) Electrofishing; (3) Net fishing; (4) Visual observation; (5) Ditch clearing; (6) Other. 

(15): If the reference type is ‘Publication’, a publication must be selected from a reference list generated from a literature management system programmed in BDW in accordance with the APA format. 

### Quality control

All data integrated into the GfI-Fischartenatlas database and published through the GfI-Fischartenatlas portal have been approved by the editorial team. Occurrence records from citable literature are directly transferred. However, occurrence records from the reference types "own notification" (i.e. from registered users) or "personal communication" (submitted to the editorial team by external parties) undergo an additional plausibility check.

## Geographic coverage

### Description

**Figure 5.**
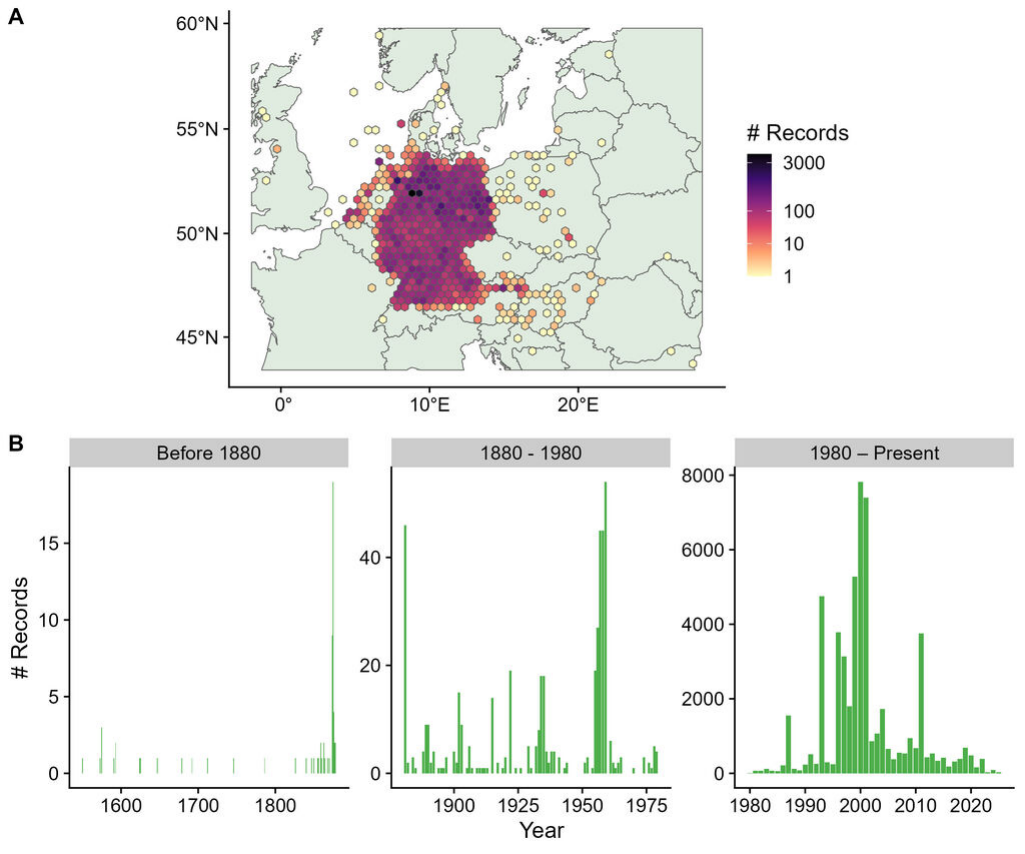
Geographic and temporal scope of the dataset. **A** Spatial distribution and number of records based on a 40 km hexagonal grid; **B** Temporal distribution as number of records per year. For better readability, the data are facetted into three time periods: Before 1880 (66 records), 1880–1980 (470 records), 1980 – Present (53485 records).

The core processing area encompasses Germany and Austria, as well as the marine areas of the Trilateral Wadden Sea Cooperation between the Netherlands, Germany and Denmark (Common Wadden Sea Secretariat 2010) and the exclusive economic zone (EEZ) of Germany. For species of particular interest, such as those listed in the Habitats Directive (Council of the European Communities 1992), occurrences from neighbouring countries are also considered on a case-by-case basis (Fig. 5).

### Coordinates

46.3 and 55.1 Latitude; 4.6 and 17.2 Longitude.

## Taxonomic coverage

### Description

Fishes and lampreys

### Taxa included

**Table:** 

Rank	Scientific Name	Common Name
class	Petromyzontida	lampreys
subclass	Elasmobranchii	sharks, rays and skates
class	Actinopterygii	ray-finned fish

## Temporal coverage

**Living time period:** From the 16th century to the present day.

## Usage licence

### Usage licence

Creative Commons Public Domain Waiver (CC-Zero)

## Data resources

### Data package title

GfI-Fischartenatlas dataset on GBIF

### Resource link

https://doi.org/10.15468/ffu3mr

### Alternative identifiers

https://www.gbif.org/dataset/f5c13912-4f12-4fd0-b1c2-0d74564a5374; https://biocase.biodiv-atlas.de/biocase/downloads/gfifish/GfI-Fischartenatlas.ABCD_2.06.zip

### Number of data sets

1

### Data set 1.

#### Data set name

GfI-Fischartenatlas dataset on GBIF

#### Data format

ABCD 

#### Character set

UTF-8

#### Download URL

https://doi.org/10.15468/ffu3mr

#### Description

The dataset is provided to GBIF through the BioCASe Provider Software installation (version 3.8.3, Anonymous (2012)) at the Leibniz Institute for the Analysis of Biodiversity Change - Museum Koenig, Bonn using the ABCD data standard (Access to Biological Collections Data).

Following the guiding rails of the FAIR principles (Wilkinson 2016), the observation data of the atlas have been mapped out to the Access To Biological Collection Data (ABCD) metadata standard which is a collaborative effort of the Biodiversity Information Standards (TDWG) community (Access to Biological Collections Data Task Group 2005). The information from the atlas database has been mapped out to 42 fields of ABCD in total, covering five domains of information on two levels of organisation). The Dataset-level metadata encompasses contact information (content and technical contacts), dataset identification (GUID, title, details and URI), intellectual property rights (licence text and URIs), ownership information (person and organisation details) and revision tracking (modification dates). On the unit-level, observation records cover four core areas of information: unit identification (UnitID, RecordURI, RecordBasis and institutional identifiers), gathering information (person names, date ranges, methods and notes), geographic coordinates (latitude, longitude and errors) and taxonomic identification (scientific names and higher taxonomic ranks). Literature references are integrated through citation titles and DOI URIs. Using this standard enables existing harvesting piplines to consume the observation data into research networks like the German Federation for Biological Data (GFBio, search.gfbio.org) and GBIF enabling a wider visibility and reuse. For a detailed overview of fields and their description, see Table 1.

## Figures and Tables

**Table 1.** Description of ABCD fields that are delivered with this dataset. Note: When downloading this dataset from GBIF, the ABCD format is transformed to the Darwin Core Format, which will result in a reduced set of fields.

Level of organisation	Domain of information	ABCD field	Description
Dataset level	Contact information	Content	Scientific contact responsible for the dataset's content (curatorial point of contact for questions about the data).
		Technical contact	Person responsible for the technical operation and maintenance of the data provider.
	Dataset identification	GUID	Globally unique dataset identifier (GUID) used to reference the dataset across systems.
		Title	Human-readable title of the dataset (with language attribute @language = de-DE).
		Details	Free-text abstract describing the scope, content and origin of the dataset.
		URI	Resolvable URL of the dataset's landing/home page.
	Intellectual property rights	Licence text	Full licence statement and additional terms of use.
		URIs	Resolvable URL of the applied licence.
	Ownership information	Person	Full name of the dataset owner together with an associated web address.
		Organisation details	Details of the owning organisation.
	Revision tracking	Modification dates	ISO xs:dateTime of the latest modification of the dataset.
Unit level	Unit identification	UnitID	Local record ID within the dataset.
		Record URI	Stable URL pointing to the public species detail page on biodiv-atlas.de.
		RecordBasis	The nature of the evidence underlying the record (e.g. HumanObservation).
		Institutional identifiers	Code of the holding institution (GfI) and code of the source dataset (gfifish).
	Gathering information	Person names	Name of the observer or, for literature-based records, the citation of the source. Personal observations without explicit consent are replaced by "Data withheld" in accordance with the GDPR.
		Date ranges	ISO 8601 start and end date/time of the sampling event.
		Methods	Method used to obtain the observation (e.g. electrofishing, netting, sight observation, literature record).
		Notes	Free-text remarks concerning the locality and/or the observation; omitted when empty.
		Country	Country name and ISO 3166-1 alpha-2 code, derived from the observation coordinates via Natural Earth boundaries, with a nearest-country fallback for offshore and marine points (North Sea, Baltic, Wadden Sea).
		Geodetic datum	Geodetic reference system to which the latitude/longitude values refer; always WGS84 (EPSG:4326).
	Geographic coordinates	Latitude	Decimal latitude in WGS84 (EPSG:4326), rounded to 6 decimal places.
		Longitude	Decimal longitude in WGS84 (EPSG:4326), rounded to 6 decimal places.
		Errors	Positional uncertainty in metres and the geodetic datum to which the coordinates refer (always WGS84).
	Taxonomic identification	Scientific names	Full Latin name of the identified species, together with the zoological authorship (author and year of original description).
		Higher taxonomic ranks	Higher-rank classification of the identified species (e.g. family, order, class) with the corresponding rank label.
		Scientific name authorship	Zoological authorship of the species: name of the original describer and year of description.
	Literature information	Citation title	Formatted citation of the originating literature reference, where applicable.
		DOI URIs	Resolvable DOI link of the cited reference, or the publication URL where no DOI is available.
